# Supplementary material for: Biomarkers for Outcome in Metastatic Melanoma in First Line Treatment with Immune Checkpoint Inhibitors
Source: Biomedicines. 2023 Mar 1;11(3):749. doi: 10.3390/biomedicines11030749 (PMC10044937; doi:10.3390/biomedicines11030749)
Supplement: Supplementary file 1 [file biomedicines-11-00749-s001.zip › biomedicines-2249098-supplementary.pdf]

Supplementary Material

Biomarkers for Outcome in Metastatic Melanoma in First Line Treatment with Immune Checkpoint Inhibitors

Tanja Mesti [1,2], Cvetka Grašič Kuhar [1,2] and Janja Ocvirk [1,2,*]

[1] Institute of Oncology Ljubljana, Zaloška 2, 1000 Ljubljana, Slovenia
[2] Faculty of Medicine, University of Ljubljana, Korytkova Ulica 2, 1000 Ljubljana, Slovenia
* Correspondence: jocvirk@onko-i.si

Supplementary Figures

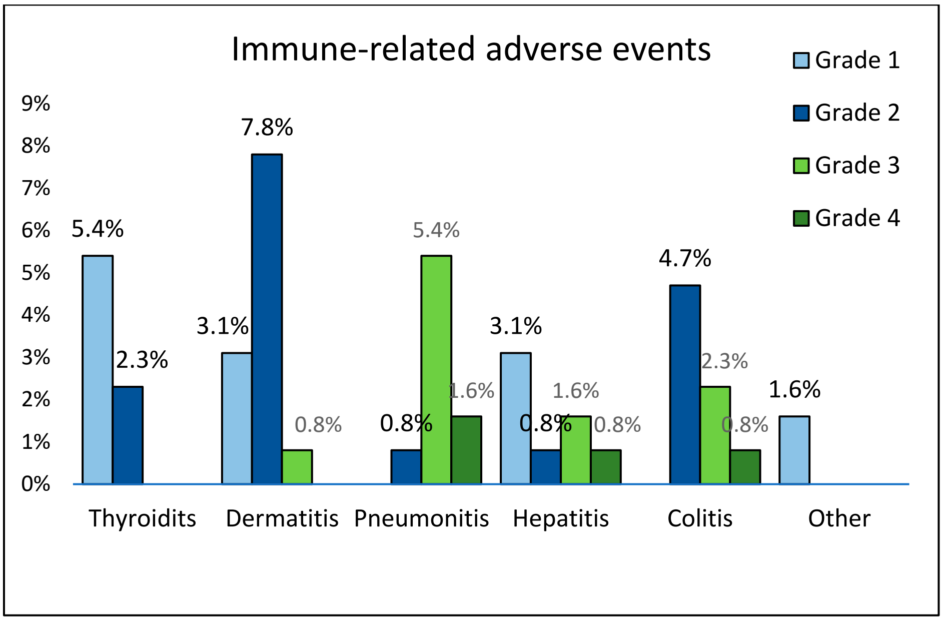

Supplementary Figure S1. Distribution of immune related adverse effects according to organ system and their grades. irAE – immune related adverse events.

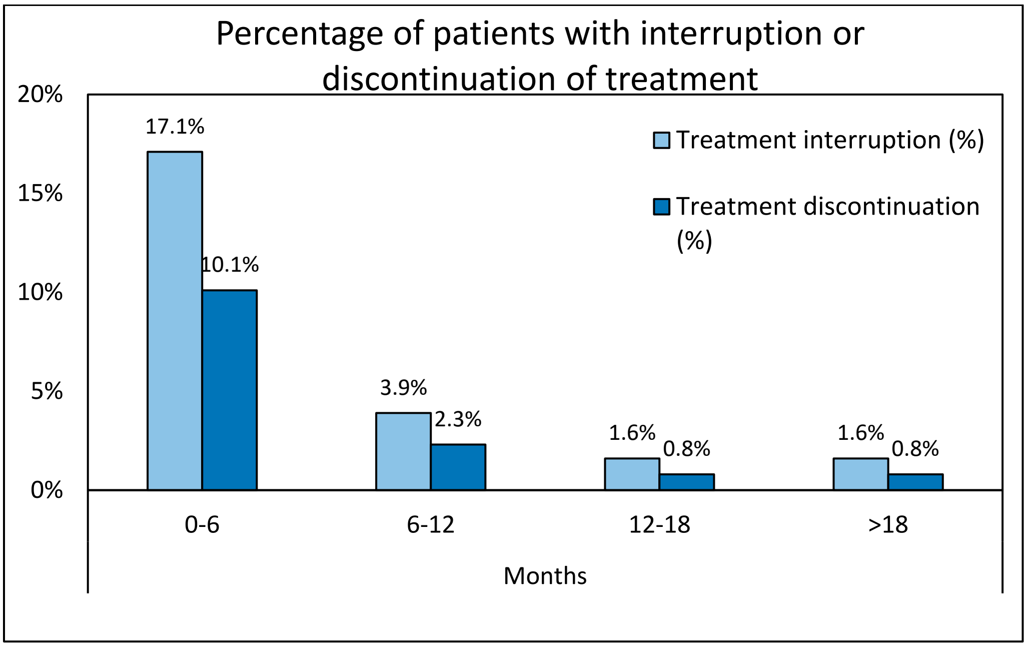

Supplementary Figure S2. Time course of treatment interruption or treatment discontinuation due to immune related adverse events.

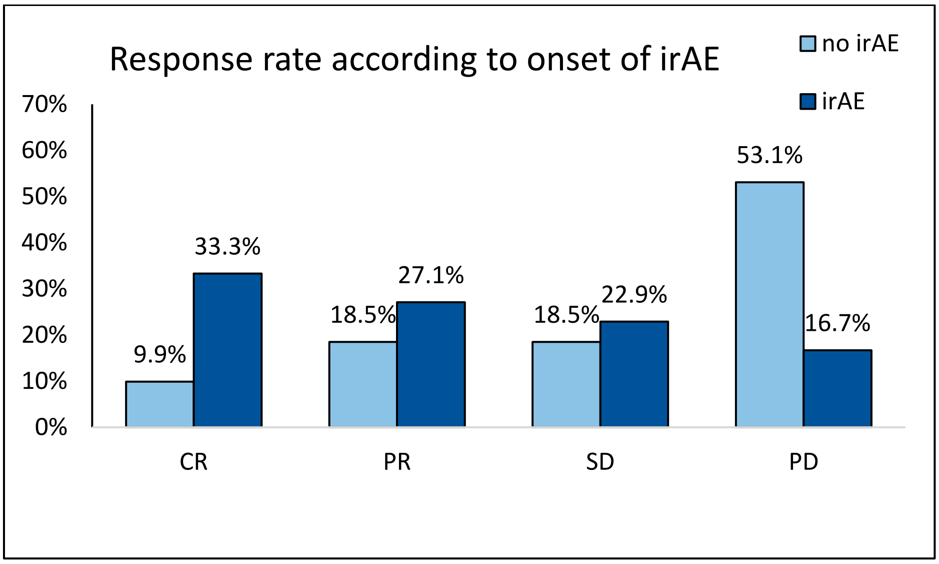

Supplementary Figure S3. Response rate distribution in patients with metastatic melanoma treated with first line immune checkpoint inhibitors according onset immune related adverse events (irAE); ($p<0.001$). CR – complete response, PR – partial response, SD – stabile disease, PD – progression disease.
